# CAREGIVING PUBLIC HEALTH SURVEILLANCE: EXPERT-GUIDED COLLABORATION FOR BRFSS MODULE REVISION

**DOI:** 10.1093/geroni/igad104.2832

**Published:** 2023-12-21

**Authors:** John Shean, Benjamin Olivari

**Affiliations:** Alzheimer’s Association, Chicago, Illinois, United States; CDC, Atlanta, Georgia, United States

## Abstract

The Behavioral Risk Factor Surveillance System (BRFSS) Caregiver Optional Module collects population-level data on caregiving prevalence, burdens faced by caregivers, and demographic information about care recipients. Developed in 2009, periodic reviews ensure the module remains current, data collected remain actionable, and encourage uptake of the module by state health agencies. In 2022, the Alzheimer’s Association and the Centers for Disease Control and Prevention’s Alzheimer’s Disease Program formed an expert workgroup to examine the ongoing value and relevance of the module and to propose improvements. The thirteen-member workgroup included academic researchers, caregiving experts, health promotion directors, epidemiologists, survey methodologists, policy analysts, and BRFSS coordinators. Over four months, the workgroup used a consensus-building process to identify areas of agreement and disagreement which informed discussion. Among many factors, the workgroup assessed the existing module for accuracy and utility. Discussion centered on utilizing plain language in the module’s questions, streamlining response options, and ensuring collection of a full scope of burden faced by caregivers, including caregivers of persons with dementia. Persons living with cognitive impairment and their caregivers were consulted during the process to ensure phrasing reflected their lived experience. The result was consensus on suggested revisions to the Caregiver Module, which was submitted to CDC for review and approval in April 2023. The revised module will be featured in this session. The robust process, involving a variety of stakeholders and perspectives, can serve as an efficient and effective model for ensuring sustainability and data-informed action using population health surveillance.

